# Immune response induced by oral administration with a *Saccharomyces cerevisiae*-based SARS-CoV-2 vaccine in mice

**DOI:** 10.1186/s12934-021-01584-5

**Published:** 2021-05-05

**Authors:** Tong Gao, Yi Ren, Shuangqin Li, Xin Lu, Han Lei

**Affiliations:** College of Medicine, Southwest Jiaotong University, Chengdu, 610031 Sichuan China

**Keywords:** EBY100/pYD1-RBD, Immune response, Universal technology platform

## Abstract

**Background:**

The global pandemic of coronavirus disease 2019 (COVID-19) caused by severe acute respiratory syndrome coronavirus 2 (SARS-CoV-2) highlights the need to develop safe and effective vaccines with a top priority. Multiple vaccine candidates are under development, and several vaccines are currently available. Efforts need to be undertaken to counter the threat of the global COVID-19 pandemic.

**Results:**

We generated a *Saccharomyces cerevisiae* (*S. cerevisiae*)-based SARS-CoV-2 vaccine, EBY100/pYD1-RBD, in which the full-length receptor binding domain (RBD) of the spike protein of SARS-CoV-2 was expressed on the surface of yeast. Mice vaccinated orally with unadjuvanted EBY100/pYD1-RBD could produce significant humoral and mucosal responses as well as robust cellular immune responses. Notably, EBY100/pYD1-RBD elicited a mixed Th1/Th2-type cellular immune response with a Th1-biased immune response in a mouse model.

**Conclusions:**

Our findings highlight the importance of the RBD as a key target to design and develop vaccines against SARS-CoV-2 and provide evidence of oral administration of a *S. cerevisiae*-based SARS-CoV-2 vaccine eliciting significant immune responses. Most importantly, the *S. cerevisiae* surface display system can serve as a universal technology platform and be applied to develop other oral viral or bacterial vaccines.

## Background

Severe acute respiratory syndrome coronavirus 2 (SARS-CoV-2), causing coronavirus disease 2019 (COVID-19), is rapidly causing a global pandemic and leading to widespread social and economic disruptions [1]. According to statistical data from the World Health Organization (WHO), as of 22 January 2021, there have been 96,267,473 confirmed cases of COVID-19, including 2,082,745 deaths [2]. Unfortunately, new infections and deaths are still increasing worldwide [3]. More alarmingly, a new and significantly more infectious variant of COVID-19 originating in the United Kingdom has been identified [4] and poses a great potential threat to epidemic prevention and control. There is no doubt that a safe and effective vaccine is urgently needed to prevent and control the current COVID-19 outbreaks.

SARS-CoV-2 belongs to the genus *Betacoronavirus* in the family *Coronaviridae*, which is a single-stranded RNA virus encoding four major structural proteins, namely, spike (S), envelope (E), nucleocapsid (N) and membrane (M) proteins [5, 6]. Of these proteins, the S protein is mainly involved in viral invasion and consists of S1 and S2 subunits [7]. Further, the receptor binding domain (RBD) at the C-terminus of the S1 subunit is responsible for recognizing angiotensin-converting enzyme 2 (ACE2) on the surface of human respiratory tract cells [8]. The S2 subunit participates in membrane fusion between the virus and host cell, which is required for virus entry [9]. Therefore, the S protein and RBD are the main targets for designing and developing COVID-19 vaccines [10–14].

Various COVID-19 vaccines, such as nucleic acid vaccines, virus vectored vaccines, virus-like particle vaccines, whole-inactivated vaccines, live attenuated vaccines and protein subunit vaccines, have been developed in time to prevent repeated or continuous epidemics [1, 15–23]. Of these, BioNTech/Pfizer Inc.’s mRNA vaccine, the Oxford University vaccine candidate AZD1222 and China National Bio-tech Group (CNBG)’s inactivated vaccine have already been approved for priority in high-risk populations.

*Saccharomyces cerevisiae* is an ideal eukaryotic host and has been engineered to express bacterial, viral or tumour antigens [24]. Oral vaccination of *S. cerevisiae* surface-displayed vaccine candidates has shown promise for preventing bacterial or viral infection [25–27]. In our previous studies, oral administration of H7N9 HA on the surface of *S. cerevisiae* provided 100% protective efficacy against homologous virus challenge in a mouse model [25]. Furthermore, the immunogenicity of UreB or VacA of *Helicobacter pylori* (*H. pylori*) expressed on the *S. cerevisiae* surface was also investigated [26]. Therefore, an *S. cerevisiae*-based manufacturing platform can serve as an alternative approach for the development of oral vaccine candidates in a timely manner.

In the present study, we generated EBY100/pYD1-RBD in which the RBD of SARS-CoV-2 was expressed on the *S. cerevisiae* surface. Western blotting, immunofluorescence assays and flow cytometric analysis were performed to confirm the location of RBD expression. Further, we demonstrated that oral vaccination with EBY100/pYD1-RBD could induce a significant humoral immune response, mucosal immune response and cell-mediated immune response in a mouse model. Hence, oral administration of EBY100/pYD1-RBD could be an attractive COVID-19 vaccine formulation and candidate for further investigation in a large animal model.

## Methods

### Molecular construction of EBY100/pYD1-RBD

The RBD gene (591 bp) was PCR amplified with primers F-1 (5′-CTAGCTAGCCCTAATATTACAAACTT-3′) and R-1 (5′-CCGGAATTCTTATGGTGCATGTAGAAGTTC-3′) based on the S gene sequence of SARS-CoV-2 (GenBank accession number: MN908947), in which NheI and EcoRI restriction enzyme sites were underlined, respectively. The PCR product was subcloned into the shuttle plasmid pYD1, and the recombinant plasmid pYD1-RBD was then transformed into competent *Escherichia coli* (*E. coli*) DH5α. Further recombinant plasmid pYD1-RBD from *E. coli* DH5α was electroporated into competent *S. cerevisiae* EBY100, and the positive clone EBY100/pYD1-RBD was screened by *Trp*^−^selective solid medium that contained 0.67% yeast nitrogen base (YNB) without amino acids, 2% glucose, 0.01% leucine, 2% agar, and 1 M sorbitol at 30 °C for 72 h.

An EBY100/pYD1-RBD clone after sequencing was cultured in 3 mL of YNB-CAA (20 g/L dextrose, 6.7 g/L YNB without amino acids, 13.61 g/L Na_2_HPO_4_, 7.48 g/L NaH_2_PO_4_ and 5 g/L casamino acids) overnight at 30 °C and expressed in YNB-CAA medium where dextrose was replaced by 20 g/L galactose at 20 °C at 72 h post-induction. EBY100/pYD1 was used as a negative control for all subsequent tests.

### Western blot analysis

Two OD_600nm_ EBY100/pYD1-RBD pellets were boiled for 10 min with 50 µL of 6 × SDS loading buffer supplemented with 0.6 mol/L Dithiothreitol (DTT). Then, the supernatants were collected and run on a 10% SDS-PAGE gel (Bio-Rad, USA). The gel was transferred to a 0.45 μm polyvinylidene difluoride (PVDF) membrane (Bio-Rad, USA). The membrane was incubated with 1:1000 diluted rabbit anti-RBD antibody (Sino Biological, China) at 4 °C for 4 h after blocking with 5% nonfat milk at room temperature for 1 h, followed by 1:5000 diluted horseradish peroxidase (HRP)-conjugated goat anti-rabbit IgG (Abcam, USA), which was used as the secondary antibody at room temperature for 1 h. Then, the membrane was reacted with ECL chemiluminescent reagent (Bio-Rad, USA) in the dark for 5 min. Finally, the blot signal was imaged using a ChemiDoc XRS System (Bio-Rad, USA). Precision Plus Protein™ WesternC™ (Bio-Rad, USA) was used as a protein marker.

### Immunofluorescence assay and flow cytometric analysis

Two OD_600nm_ of EBY100/pYD1-RBD pellets were incubated with 1:1000 diluted rabbit anti-RBD antibody (Sino Biological, China) at 4 °C for 1 h, followed by goat anti-rabbit IgG-FITC conjugates (Sigma, USA) at a dilution of 1:5000 for 40 min at room temperature, and resuspended in 500 µL of sterile PBS. Finally, EBY100/pYD1-RBD pellets were imaged for immunofluorescence assay (Nikon, Japan) and analyzed by flow cytometry analysis (BD FacsCalibur, USA).

### Vaccine preparation

EBY100/pYD1-RBD cells were treated by heat inactivation at 60 °C for 1 h. The final concentration of EBY100/pYD1-RBD was adjusted to 1.0 OD_600nm_/µL.

### Monoclonal antibody (mAb) binding to EBY100/pYD1-RBD

The binding affinity between EBY100/pYD1-RBD before or after heat inactivation and neutralizing anti-RBD mAb was detected by ELISA. Briefly, EBY100/pYD1-RBD was coated overnight at 4 °C. The ELISA plate was blocked with 2% TBST for 2 h at 37 °C, and incubated at 37 °C with serially diluted neutralizing mAb (Sino Biological, China). The plate was washed and then incubated with 1:5000 diluted HRP-conjugated goat anti-mouse IgG antibody (Abcam, USA) for 1 h at 37  °C. After washing, the plate was further incubated with 3,3′,5,5′-tetramethylbenzidine (TMB) substrate (Sigma, St. Louis, MO, USA), and the reaction was stopped with 1 mol/L H_2_SO_4_. Absorbance at 450 nm (A450) was measured by ELISA microplate reader (BioTek, USA).

### Inhibition of EBY100/pYD1-RBD binding to human ACE2

Inhibition of binding between human ACE2 (hACE2) and EBY100/pYD1-RBD before or after heat inactivation was detected by ELSA. Briefly, neutralizing mAbs (Sino Biological, China) were serially diluted, mixed with EBY100/pYD1-RBD before or after heat inactivation and incubated for 30 min at 37 °C. The mixture was then added to and the ELISA plate pre-coated at 4 °C with 2 mg/mL hACE2 (Sino Biological, China) in PBS. The plate was washed and RBD binding was revealed using 1:5000 diluted HRP-conjugated goat anti-mouse IgG antibody (Abcam, USA). After washing, TMB substrate was added and the plate was read at 450nm. The percentage of inhibition was calculated as follows: (1– [(OD sample – OD negative control)/(OD positive control – OD negat ive control]) ×100. Sample: EBY100/pYD1-RBD before or after heat inactivation; Negative control: EBY100/pYD1 before or after heat inactivation; Positive control: Purified RBD protein.

### Animals, oral immunization and sample collection

Eight-week-old female BALB/c mice were purchased from Chengdu Dasuo Experimental Animal Co., Ltd. (Chengdu, China) and housed in the specific pathogen-free (SPF) animal center of Southwest Jiaotong University. The mice (n = 15 per group) were orally administered 150 µL of EBY100/pYD1-RBD on days 1 and 2 for prime immunization and days 14 and 15 for boost immunization. The same dosage of EBY100/pYD1 or PBS was used as a control.

Blood samples (n = 10 per group) and feces (n = 10 per group) were collected from the vaccinated mice at day 12 and day 25 after the initial immunization.

Sera were isolated by centrifugation at 2000*g* for 10 min at room temperature and then stored at − 20 °C until use. Fecal pellets (30 mg/mouse) were suspended in 300 µL of sterile PBS. The fecal supernatants were collected by centrifugation at 24,000*g* for 8 min and stored at − 20 °C until use.

All animal studies were in accordance with the Guidelines for Use and Care of Experimental Animals and approved by the Animal Committee of the Institute of Southwest Jiaotong University.

### Enzyme‐linked immunosorbent assay (ELISA)

The RBD-specific antibodies regarding IgG titers in the sera and IgA titers in the feces were determined by ELISA. Recombinant RBD protein (Sino Biological, China) was used to coat flat-bottom 96-well plates (Costar, Corning, USA) at a final concentration of 2 µg/mL in carbonate coating buffer (pH 9.6) at 4 °C overnight. Plates were washed three times with Tris-NaCl buffer (TBS) containing 0.05% Tween 20 (TBST) and blocked with 1% BSA in TBST at room temperature for 2 h.

To determine the IgG titer, serially diluted sera were added and incubated at 37 °C for 1 h, and then the plates were washed three times with TBST. Then, 1:5000 diluted biotinylated goat anti-mouse IgG (R&D Systems, USA) was added to the wells (100 µL), and the plates were incubated at 37 °C for 1 h and washed three times with TBST, followed by the addition of 1:1000 diluted alkaline phosphatase (AP)-conjugated streptavidin (R&D Systems, USA) (100 µL) at room temperature for 1 h. Finally, the plates were washed three times with TBST and developed with a pNPP phosphatase substrate (MP Biomedicals, USA) in the dark for 25 min. The reaction was stopped with 2 mol/L NaOH (50 µL). The absorbance was measured on a microplate reader (Bio-Tek, USA) at 405 nm. The IgG titers were expressed as the reciprocal of the highest serum dilution that yielded an OD_405 nm_ value greater than twice the mean plus one standard deviation of the control samples.

To determine secretory IgA titers, the fecal supernatant was added and incubated at 37 °C for 1 h. The plates were washed three times with TBST, and 1:20,000 diluted HRP-conjugated goat anti-mouse IgA was added to the wells (100 µL). After incubation at 37 °C for 1 h and washing three times with TBST, 3,3′,5,5′-tetramethylbenzidine (TMB) was used as a substrate (100 µL), and the reaction was stopped with 0.5 M H_2_SO_4_ (50 µL). The absorbance was measured on a microplate reader (Bio-Tek, USA) at 450 nm. The IgA titers are presented as the OD_450 nm_ value per 10 mg of feces.

### Lymphocyte proliferation assay

Splenocytes were isolated from the vaccinated mice (n = 5 per group) on days 12 and 25 after the initial vaccination (2 × 10^5^ cells/well) and cultured in RPMI 1640 medium supplied with 10% (v/v) FBS, 100 U/mL penicillin, 100 µg/mL streptomycin, and 1 mM pyruvate. Recombinant RBD protein (2 µg/mL, Sino Biological, China) was used as a stimulus. After 36 h of incubation, 10 µL of the cell counting kit-8 (CCK-8) solution (Dojindo Laboratories, Kumamoto, Japan) was added to each well. The subsequent procedures were performed according to the manufacturer’s instructions. The plates were measured at 450 nm using a microplate reader (Bio-Tek, USA).

### Expression of cytokine

To investigate cell-mediated immune responses, the expression levels of interferon gamma (IFN-γ) and interleukin-4 (IL-4) were analyzed using ELISA kits (R&D Systems, USA) according to the manufacturer’s instructions. Briefly, splenocytes (1.0 × 10^6^ cells/well) described above were cultured in medium containing 10% (v/v) FBS, 100 U/mL penicillin, 100 µg/mL streptomycin, 1 mM pyruvate, 50 µM β-mercaptoethanol and 20 U/mL IL-2 and stimulated with 2 µg/mL recombinant RBD protein (Sino Biological, China) for 72 h in a humidified incubator at 37 °C with 5% CO_2_. The supernatants were collected for ELISA experiments to analyze the expression levels of cytokines.

The phenotypes of these cultured lymphocytes were determined by flow cytometry. In brief, these cultured lymphocytes were washed with sterile PBS and then stained for 30 min at 4 °C with monoclonal anti-CD8 and anti-CD4 antibodies. Afterwards, cells were fixed and permeabilized to facilitate intracellular staining with anti-IFN-γ and anti-IL-4 antibodies.

### SARS-CoV-2 pseudovirus neutralization assay

SARS-CoV-2 pseudotyped virus was used to test the neutralizing activities of sera from the vaccinated mice as previously described [20]. Briefly, HEK293T cells were transfected with a plasmid encoding codon-optimized SARS-CoV-2 S protein (Genscript, NJ, USA) and pNL4-3. Luc.R-E- plasmid (BioVector NTCC Inc. Beijing, China) at a 1:1 ratio. Forty-eight hours post transfection, the culture supernatants containing luminescence-expressing pseudoviruses were collected, sterile-filtered (Millipore Sigma), and aliquoted for storage at − 80 °C until use. Sera were serially diluted twofold starting at a 1:8 dilution after heat inactivation for 20 min at 56 °C and incubated with a fixed amount of SARS-Cov-2 pseudotyped virus for 60 min at 30 °C. HEK293T cells stably expressing hACE2 were added. After 72 h post infection, the relative luminescence units (RLU) were measured on a plate reader (Biotek, USA) using the Bright-Glo Luciferase Assay System (Promega, USA), according to the manufacturer’s recommendations. Neutralization titers (ID_50_) were calculated as the serum dilution at which RLU were reduced by 50% compared with RLU in virus control wells after subtraction of background RLU in cell control wells. The ID_50_ value less than 40 was considered no significance.

### Statistical analysis

The data are presented as the means ± standard deviation (SD). Statistical analysis was performed by one-way analysis of variance (ANOVA) with post hoc multiple comparison analysis. The statistical significance was expressed as *p* < 0.05. *p < 0.05, **p < 0.01.

## Results

### Construction and expression analysis of EBY100/pYD1-RBD

**Fig. 1:**
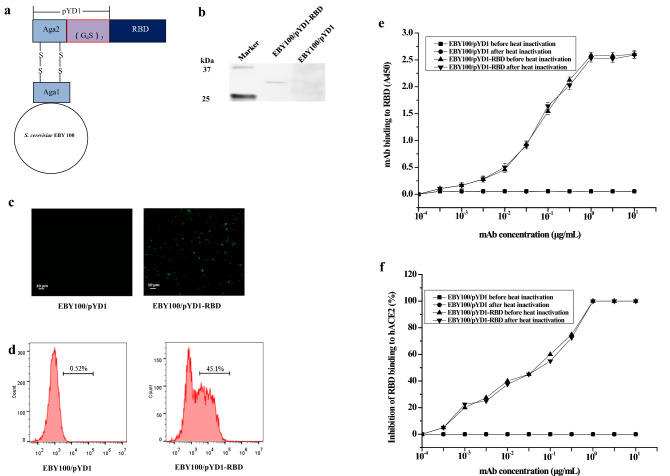
Schematic diagram and expression analysis of EBY100/pYD1-RBD. **a** Schematic model of EBY100/pYD1-RBD. The RBD protein was fused with Aga2, which was expressed by pYD1 as the subunit of the a-agglutinin receptor via the Gly-Ser linker. Further, Aga2 was then bound to Aga1, which is the subunit of yeast, through two disulfide bonds. **b** Western blot analysis. **c** Immunofluorescence assay. EBY100/pYD1 (left side); EBY100/pYD1-RBD (right side). (Magnification: ×400). **d** Flow cytometric analysis. EBY100/pYD1 (left side); EBY100/pYD1-RBD (right side, 45.1%). 10,000 cells were counted. The results from Western blot, immunofluorescence microscopy assay and flow cytometric analysis are representative of three independent experiments. **e** mAb-mediated binding to EBY100/pYD1-RBD detected by ELISA. The data are presented as the means ± SDs. **f** mAb-mediated inhibition of EBY100/pYD1-RBD binding to hACE2 analyzed by ELISA

The RBD gene of the spike protein of SARS-CoV-2 was inserted into plasmid pYD1, and then the RBD protein was fused with Aga2, which was expressed by pYD1 as the subunit of the a-agglutinin receptor via the Gly-Ser linker. Further, Aga2 was then bound to Aga1, which is the subunit of yeast, through two disulfide bonds (Fig. 1a).

Western blot analysis was used to identify the expression of RBD protein in EBY100/pYD1-RBD. As shown in Fig. 1b, there was no specific band obtained in EBY100/pYD1, whereas an expected band appeared in EBY100/pYD1-RBD, whose molecular weight is approximately 32 kDa and consists of Aga2 (10 kDa) and RBD (22 kDa).

**Fig. 2:**
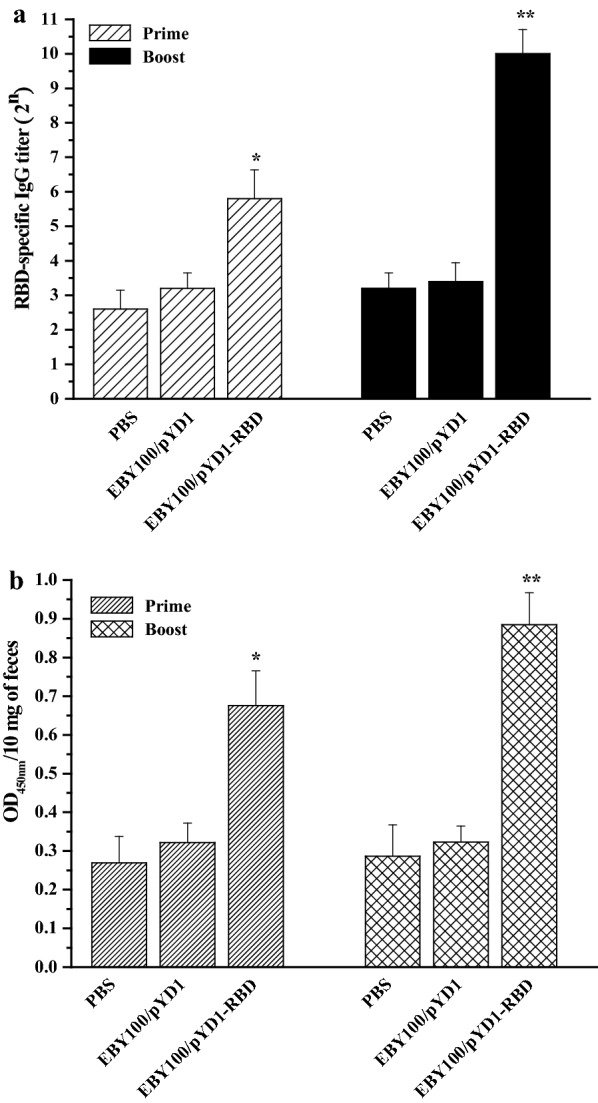
Humoral and mucosal immune responses elicited by EBY100/pYD1-RBD. **a** RBD-specific serum IgG titers (n = 10 mice/group). **b** Secretory IgA in the feces (n = 10 mice/group). The data shown are the means ± SDs of three technical replicates per measurement, and the asterisks indicate significant differences compared with the PBS- and EBY100/pYD1-vaccinated controls. *p < 0.05, **p < 0.01

Furthermore, immunofluorescence assays and flow cytometric analysis were performed to further confirm the location of the RBD. As shown in Fig. 2c and d, compared with EBY100/pYD1, positive signals strongly appeared in EBY100/pYD1-RBD treated directly with anti-RBD antibody, which corresponded to the positive rate (45.1%). These results demonstrate that the RBD protein is expressed on the surface of EBY100.

### Binding affinity between mAb and EBY100/pYD1-RBD

The binding affinity of mAb with EBY100/pYD1-RBD before or after heat inactivation was determined by ELISA. As shown in Fig. 1e, EBY100/pYD1-RBD before or after heat inactivation showed a similar binding affinity and bound significantly to mAb, whereas the binding affinity between mAb and EBY100/pYD1 was indistinguishable from background. The results demonstrated that the binding affinity of EBY100/pYD1-RBD before or after heat inactivation had no significant difference.

### Blockade of EBY100/pYD1-RBD binding to hACE2

We further measured the inhibitory activity of EBY100/pYD1 before or after heat inactivation binding to hACE2. As shown in Fig. 1f, EBY100/pYD1 before or after heat inactivation had no inhibitory activity. By contrast, neutralizing mAb could effectively block the binding of EBY100/pYD1-RBD before or after heat inactivation to the hACE2.

### Humoral and mucosal immune responses induced by EBY100/pYD1-RBD

To assess the humoral and mucosal immune responses induced by EBY100/pYD1-RBD, sera and feces were collected from the orally vaccinated mice on days 14 and 28 after the primary immunization. The IgG titers in the sera and the OD_450 nm_ values of IgA in the feces were determined by ELISA. As shown in Fig. 2a, the titers of RBD-specific serum IgG antibodies induced by EBY100/pYD1-RBD were higher than those induced by EBY100/pYD1 or PBS during the primary immunization, and the IgG titers in the PBS group, EBY100/pYD1 group and EBY100/pYD1-RBD group were 2^2.6 ± 0.55^, 2^3.2 ± 045^ and 2^5.8 ± 0.84^, respectively. A significantly higher IgG titer elicited by EBY100/pYD1-RBD reached 2^10 ± 0.71^ compared with PBS or EBY100/pYD1 at post-boost immunization.

We also investigated the mucosal immune response induced by EBY100/pYD1-RBD. As shown in Fig. 2b, the OD_450 nm_ values of IgA antibodies in the PBS group, EBY100/pYD1 group and EBY100/pYD1-RBD group were 0.269 ± 0.069, 0.322 ± 0.051 and 0.676 ± 0.089, respectively, at day 14. A significant increase in the OD_450 nm_ value was shown in the EBY100/pYD1-RBD group, which was 0.885 ± 0.083, whereas the OD_450 nm_ values in the PBS group or EBY100/pYD1 group were not significantly changed at day 28.

These data suggest that oral administration of EBY100/pYD1-RBD can elicit significant humoral and mucosal immune responses during prime-boost immunization.

### Assessment of T cell proliferation

**Fig. 3:**
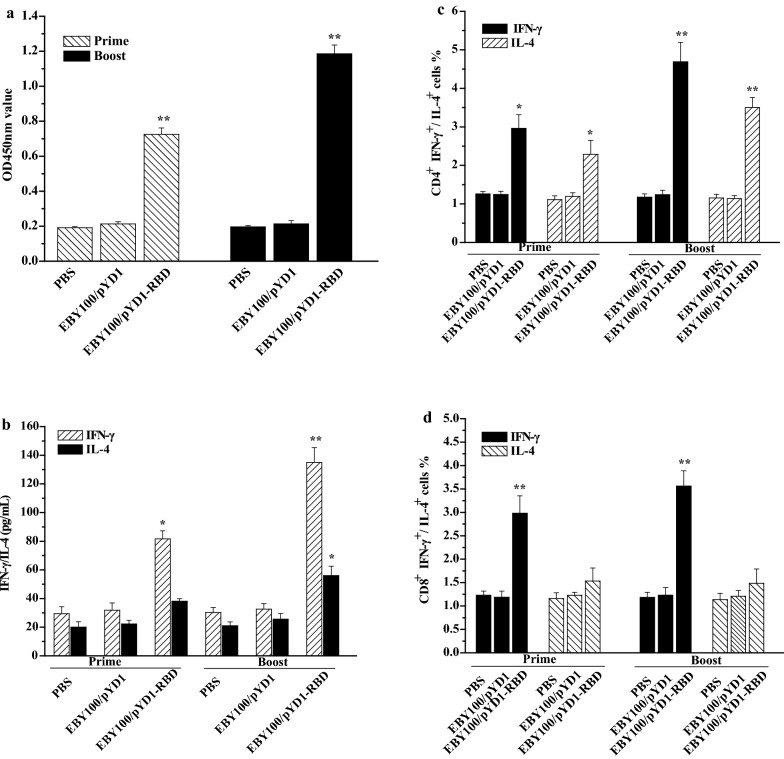
Cellular immune responses induced by EBY100/pYD1-RBD. **a** T cell proliferation. **b** The expression levels of IFN-γ and IL-4. **c** CD4^+^ cells mediated the secretions of IFN-γ and IL-4. **d** CD8^+^ cells mediated the secretions of IFN-γ and IL-4. The data are represented as the means ± SDs. Similar results were obtained in three independent experiments. The asterisks indicate significant differences compared with the PBS- and EBY100/pYD1-vaccinated controls. *p ˂ 0.05, **p ˂ 0.01

To assess T cell proliferation, splenocytes were harvested from the vaccinated mice and stimulated with the RBD protein. After 36 h of incubation, T cell proliferation was measured by a CCK-8 kit. Compared with the control groups (PBS and EBY100/pYD1), the EBY100/pYD1-RBD group exhibited rapid and significant proliferation of T cells (Fig. 3a), and the OD_450 nm_ values were 0.725 ± 0.038 and 1.186 ± 0.049 at day 14 and day 28, respectively. These results reveal that EBY100/pYD1-RBD can elicit T cell proliferation and immune responses.

### IFN-γ and IL-4 cytokine expression induced by EBY100/pYD1-RBD

To further identify the potential memory lymphocytes against the recombinant RBD protein, we detected the secretion levels of IFN-γ and IL-4. As shown in Fig. 3b, compared with the PBS and EBY100/pYD1 controls, increased levels of IFN-γ and IL-4 in the EBY100/pYD1-RBD group were observed when stimulated with the RBD protein at prime-boost immunization; moreover, the secretion level of IFN-γ was higher than the IL-4 secretion level. Furthermore, we also evaluated the numbers of memory lymphocytes, CD4^+^ IFN-γ^+^, CD8^+^ IFN-γ^+^ and CD4^+^ IL-4^+^ that were increased in the mice orally administered EBY100/pYD1-RBD (Fig. 3c, d). These results indicate that EBY100/pYD1-RBD can induce significant cellular cell immune responses and increase Th1 bias in memory lymphocytes.

### Neutralization activity induced by EBY100/pYD1-RBD

**Fig. 4:**
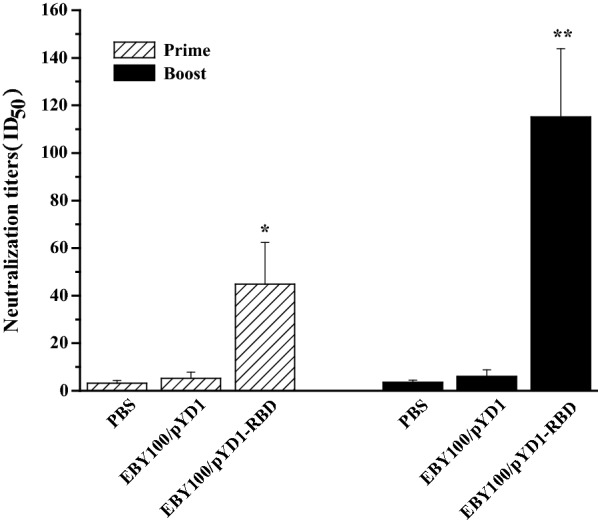
SARS-CoV-2 pseudovirus neutralization assay. Neutralizing antibody titers were measured after oral administration of EBY100/pYD1-RBD. Sera (n = 10 mice/group) were collected on day 14 and day 28 after the first immunization and serial dilutions were incubated with a pseudovirus displaying the SARA-CoV-2 Spike and co-incubated with ACE2- HEK293T cells. The data are represented as the means ± SDs. The asterisks indicate significant differences compared with the PBS- and EBY100/pYD1-vaccinated controls. *p ˂ 0.05, **p ˂ 0.01

The sera isolated from the vaccinated mice were also analyzed by SARS-CoV-2 pseudovirus neutralization assay. As shown in Fig. 4, there were no significant neutralizing antibodies detected in the PBS or EBY100/pYD1 group. By contrast, the neutralization titers (ID_50_) in the EBY100/pYD1-RBD group were 44.8 ± 17.527 and 115.2 ± 28.62 at day 14 and day 28, respectively. These data highlight that mice orally vaccinated with EBY100/pYD1-RBD after the prime-boost immunization can induce the significant levels of neutralizing antibodies against SARS-CoV-2 pseudovirus infection.

## Discussion

Because of the high transmissibility of SARS-CoV-2 and the high rate of morbidity and mortality associated with COVID-19, vaccines are likely the only public health measure to fight a major pandemic. Currently, mRNA-1273, AZD1222, BNT162b1 and inactivated COVID-19 vaccines have been approved with high priority for mass vaccination of the general public. Furthermore, multiple SARS-CoV-2 vaccine candidates, including inactivated or live attenuated virus and adjuvanted protein subunit vaccine DNA nucleic acid vaccine, are still in clinical trials [16, 28, 29]. These preclinical or clinical vaccines remain intramuscularly injected. To date, no peer-reviewed preclinical studies have been published on oral SARS-CoV-2 vaccines to date. In this study, we generated EBY100/pYD1-RBD, in which RBD was expressed on the surface of yeast, and then evaluated the immune efficacy in mice by oral administration without the use of mucosal adjuvant. Although it is a preliminary study, the fact that oral vaccination with EBY100/pYD1-RBD can induce effective humoral and mucosal immune responses, as well as cellular immune responses, is promising and encouraging.

To define a more optimized surface analysis approach, Western blotting was performed to identify the specific expression of RBD in EBY100/pYD1-RBD (Fig. 1b), which is a molecular biological method to detect antibody–protein interactions in vitro. Further, immunofluorescence assays and flow cytometric analysis assays were used to verify the expression location of the RBD (Fig. 1c, d) when EBY100/pYD1-RBD was directly labelled with a primary antibody. Collectively, Western blotting in combination with immunofluorescence assays and flow cytometric analysis can serve as a universal technology for determining the expression location of proteins. To further characterize the biological function of EBY100/pYD1-RBD, neutralizing anti-RBD mAb was used to detect the binding affinity or inhibitory activity of EBY100/pYD1-RBD, EBY100/pYD1-RBD before or after heat inactivation had no significant losses of biological activities. This finding shows that RBD displayed on the surface of *S. cerevisiae* binds to hACE2 with a high affinity, which is a good reflection of the native conformation of RBD protein.

It is generally recognized that the RBD of the spike protein can engage with receptor angiotensin-converting enzyme 2 (ACE2) on host cells [14]. The RBD domain (residues 319–545) could induce potent functional antibody responses in immunized mice, rabbits and nonhuman primates (*Macaca mulatta*) by a single injection [10]. The full-length RBD gene (591 bp) was chosen for fusion with the *S. cerevisiae* surface display plasmid pYD1, and we also confirmed that EBY100/pYD1-RBD could elicit strong humoral responses, indicating a higher serum IgG titer (Fig. 2a). Compared with the injection route, oral administration of EBY100/pYD1-RBD could induce robust mucosal immune responses, representing the OD_450nm_ value of fecal IgA (Fig. 2b), which may contribute to conferring protection against SARS-CoV-2 infection.

Cellular immune responses play a crucial role in stimulating a long duration of the humoral immune response. EBY100/pYD1-RBD induced potent CD4^+^ and CD8^+^ T cell responses. The expression level of IFN-γ, which represents Th1 cytokines, along with a high frequency of IFN-g-producing CD4^+^ and CD8^+^ T cells, was higher than the IL-4 secretion level, which represents the Th2 response (Fig. 3b–d). This result is noteworthy because EBY100/pYD1-RBD induced Th1 responses rather than Th2 responses, as a major safety consideration of EBY100/pYD1-RBD vaccine candidate design is the elicitation of a strong Th1-biased immune response instead of a Th2-biased response that might induce vaccine-associated enhanced respiratory disease [15].

Virus neutralization remains the gold standard for determining antibody efficacy. A pseudovirus neutralization assay has been developed for evaluating neutralizing antibodies against SARA-CoV-2 infection and can be performed in Biosafety Level 2 (BSL-2) containment [30]. Therefore, neutralizing titer is a key parameter to predict the immunity of vaccine candidate. We measured the neutralization titer (ID_50_) induced by EBY100/pYD1-RBD (Fig. 4), which may provide the basis for further evaluation of *S. cerevisiae*-based SARA-CoV-2 vaccine candidate against live virus challenge in vivo.

This study has several limitations. The coordination role between serum IgG and secretory IgA is still unknown. Further research will need to address the protective immunity of EBY100/pYD1-RBD in a large animal model because virus challenge is the gold-standard strategy for evaluating the effectiveness of a SARS-CoV-2 vaccine candidate. In addition, *S. cerevisiae-*based vaccines may be safer for use in humans due to their edible nature; however, certain individuals may be allergic to *S. cerevisiae*.

## Conclusions

In summary, our findings highlight the importance of the RBD protein displayed on the surface of yeast and provide a rationale for the development of an oral SARS-CoV-2 vaccine candidate that induces significant humoral and mucosal immune responses as well as robust cellular immune responses, making it technically and commercially feasible to manufacture at a global supply scale.

## Data Availability

The datasets generated and analyzed in the current study are available from the corresponding author upon reasonable request.
